# Commercially available alternatives to palm oil

**DOI:** 10.1002/lite.201600018

**Published:** 2016-04-14

**Authors:** Nils Hinrichsen

**Affiliations:** ^1^ ADM Research GmbHHamburgGermany

**Keywords:** Palm oil, Edible oils, Saturated fatty acids, Glycidoesters

## Abstract

Since several years there has been a demand for food products free of palm oil, noticeable in the Western European market. Alternatives based on liquid oils, fully hydrogenated fats, and exotic fats like shea and sal etc., have been developed by the research groups of several specialty oils and fats suppliers. This article describes the advantages and disadvantages of those products and compares them to similar products based on palm oil. It is also discussed how reasonable the replacement of palm products would be, since sustainable and 3‐MCPD/glycidolester‐reduced palm based specialty oils are also available on the market.

## Introduction

In the last few decades, palm oil has become one of the most important edible oils globally. Since it is solid at room temperature and its fractions deliver a wide range of functional melting profiles, palm oil has played an important role in the replacement of partially hydrogenated oils in food applications in Western Europe. Due to the fact that partially hydrogenated oils can contain a significant amount of *trans*‐fatty acids, which are considered as unhealthy 1, their use and application has steadily decreased since the mid‐90's. In many countries (and the number is growing) there are legal limits for the level of *trans*‐fatty acids in foods, which very often lead to the situation that partially hydrogenated fats have been replaced by palm oil or palm kernel oil based products, which both come from the oil palm tree (*Elaeis guineensis*).

However, palm oil is also under discussion as it contains a relatively high level of saturated fatty acids in comparison to liquid oils, such as rapeseed or sunflower oil, saturated fats being a health issue now for many years. Also, the cultivation of palm oil is often connected with issues of sustainability, although there is significant progress in this area via organizations such as RSPO (Round Table for Sustainable Palm Oil). The oil yield of palm oil per ha is higher than of any other oil crop, and ranges between 4 to 6 mt/year and ha (in comparison rapeseed oil yields 1.5–2.5 mt/year and ha), though palm oil has a higher potential to produce 3‐MCPD (3‐chlor‐1,2‐propandiol) and glycidolesters in the refining process compared to many other oils 2. These factors have led to negative media coverage in certain countries, such as France and Norway, making palm oil an unpopular choice. There also seems to be a growing interest in palm oil alternatives in other European countries.

Which raw materials could be used in various fat applications when palm oil is undesired? Partially hydrogenated fats would have an appropriate melting profile, but due to aforementioned *trans‐*fatty acid levels, their usage is almost eliminated in Western Europe and therefore not a viable option. Fully hydrogenated fats, liquid oils, coconut oil, cocoa butter and exotic fats like shea, sal or illipé butter remain as potential alternatives. The pros and cons will be discussed in the following paragraphs.

## Hydrogenated alternatives

**Figure 1:**
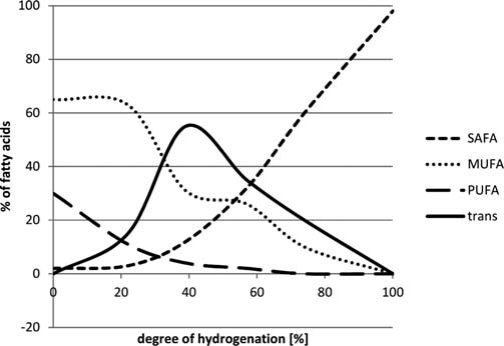
Changes of *trans‐ *and saturated fatty acid level in the hydrogenation process of a soybean oil.

Even though partially hydrogenated fats are not an option to replace palm oil in food applications, a fully hydrogenated fat could potentially be utilised. The level of *trans*‐fatty acids changes during the hydrogenation process (**Figure** 1) and at full hydrogenation (almost all double bonds saturated) a fat does not contain either unsaturated fatty acids or* trans*‐fatty acids in any significant quantity. The issue with fully hydrogenated fats (except for lauric fats, C12 and C14) is their high melting point (typically >50°C), this results in a high “solid fat content” at body temperature and therefore an unpleasant sensory experience. To achieve a melting profile that is appropriate for most food applications (almost liquid at body temperature plus functionality at lower temperatures), a fully hydrogenated oil can be blended and interesterified with various non‐hydrogenated oils to produce a functional and organoleptically acceptable end‐product.

By blending liquid oils such as rape, sunflower, soya, etc. with fully‐hydrogenated oil(s), a good baking fat blend can be achieved. The melting profile is typically flatter than that of palm oil, especially at lower temperatures. However, in the temperature range between 20 and 40°C, which is a critical area in processing of a baking fat, the melting profile is very similar to palm oil. Due to its relatively high amount of liquid oil, the blend will have a level of saturated fatty acids ca. 15% lower than palm oil, for a similar functional product.

Another option would be to blend the hydrogenated liquid oil with a lauric fat, such as coconut oil. Using coconut oil in combination with fully hydrogenated oils will produce a much steeper melting profile and a higher solid fat content at 10 and 20°C. Due to the steep melting profiles and pleasant meltdown at body temperature, such blends can give very good results in confectionary fillings. However as coconut oil and fully hydrogenated liquid oils both have a very high level of saturated fatty acids, the blend will be significantly higher in saturates than palm oil and therefore may be an issue for some customers and/or consumers.

Generally, when fully hydrogenated/saturated fats are used, the overall saturate content is dependent on the choice of additional components (either liquid or coconut oil). If liquid oils are used, the level is normally lower compared to palm oil; if coconut oil is used, the blend will have a significantly higher level of saturated fatty acids. Therefore the required steep melting profile, which is necessary, for example for confectionary fats, can typically only be achieved by choosing a blend with a high level of saturated fatty acids. For solid frying fats, margarines and bakery fat blends, where a rather flat melting profile could be applied, blends of fully hydrogenated oils with liquid oils could be used and have a relatively low level of saturated fatty acids, often even lower then palm oil itself.

Even though the above mentioned fully hydrogenated fats do not contain *trans‐*fatty acids, in many countries it is still necessary to label the process of hydrogenation on the food packaging, although the EU commission is reviewing this as part of potential “trans” legislation. If a food should be labeled as “non‐hydrogenated” and “palm oil free”, fat blends based on exotic fats, might therefore be a better solution.

## Alternatives based on exotic fats or cocoa butter

“Exotic fats” are typically fats such as shea, sal, illipé, kokum and mango kernel, in addition there are many other “exotic fats”, but these are not available in significant volumes. They all have in common a relatively high level of symmetrical triglycerides (triglycerides having a saturated‐unsaturated‐saturated configuration; such as SOS, POS, POP) and therefore need special tempering to crystallize into the desired stable form. Cocoa butter also has a high level of symmetrical triglycerides and accordingly a similar, probably even more complex, crystallization behavior, and therefore can also be used as a component in non‐palm, non‐hydro‐fats. Some exotic fats, for example shea, are not used directly, but only after fractionation with the symmetrical triglycerides being concentrated in the stearin fraction. With some other exotic fats, the triglyceride configuration is already adequate for confectionery applications without the need for fractionation.

**Figure 2:**
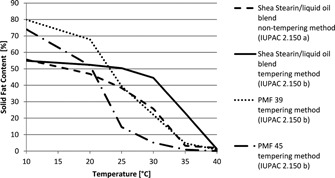
Solid fat contents of palm oil mid fractions (iodine value 39 and 45) and of a shea stearin/liquid oil blend. The results for the shea stearin/liquid oil blend are shown once measured with a non‐tempering method and once with a long‐time tempering method.

By blending liquid oils with shea stearin, fat blends can be produced that replace palm mid fractions, for example as a filling fat in confectionary applications. Still, to achieve the required stable crystal configuration, it is necessary to temper the filling, similar to chocolate. Especially at room temperature (ca. 20°C), a non‐tempered fat with a high level of symmetrical triglycerides will often be too soft. Also it will tend to recrystallize during storage and can cause fat bloom. A slight amendment of the recipe and processing conditions might still be necessary, as these blends will not have exactly the same melting profile as palm mid fractions (**Figure** 2).

Because of their high level of liquid oils, such blends will mostly have a level of saturated fatty acids, lower than the originally used palm mid fraction. In the described example, the non‐palm fat has a level of saturated fatty acids of 41% compared to a level of 59% in a palm mid fraction.

Blending exotic fats with liquid oils therefore can be a good solution to replace palm‐oil‐based fats in some confectionary applications when equipment to temper the product is available. In many cases though, the production lines are not equipped with tempering equipment and an upgrade would be costly. By interesterifying the exotic fats, the symmetrical triglycerides will be partially converted to asymmetrical triglycerides. In this way, a fat can be produced that is based on exotic fats, but does not need tempering due to the reconfiguration of the triglycerides. Interesterified exotic fats themselves typically have a high solid fat content across a broad temperature range and have a melting temperature much higher than body temperature. Used alone and without the addition of liquid oils or coconut oil, they are mostly too solid to be used in foods. Similar to fully‐hydrogenated fats, the addition of lauric fats to the interesterification blend with exotic fats gives the best results regarding the melting profile. A high solid fat content at 10 and 20°C and an almost liquid product at body temperature can be achieved. Again, the use of coconut oil in such an interesterifed product will increase the level of saturated fatty acids and result in mostly higher levels compared to palm based products.

One of the major drawbacks of non‐palm and non‐hydro fats based on shea, cocoa butter or other exotic fats is the high price of the raw materials. Even though in most cases a specialty fat is not composed exclusively from exotic fats, a replacement by a non‐palm solution is significantly more expensive than a palm based product, for the same performance. Traditionally, shea butter is used in cosmetics and pharmaceutical products, which also gives them a reputation as a non‐food oil. In addition, the availability of exotic fats is limited and the demand is high. Shea is rarely grown in plantations; the nuts are mainly collected by local people in the savannah and sold in small volumes to the processing plants. This makes the supply potentially susceptible to political unrests or other unexpected events. Other exotic fat trees such as Illipé only crop at irregular intervals.

## Liquid oils as alternatives to palm oil

A very obvious way to replace palm oil would be the usage of liquid oils like rapeseed oil, sunflower oil or soy bean oil. Due to their liquid aggregate state, they cannot replace all sorts of palm fractions in every application. Still, in applications where only small volumes of fat are used (for example bread, wafers, release agents) or where liquid fractions of palm oil (oleins) are applied, a replacement of palm by liquid oils is often possible.

**Figure 3:**
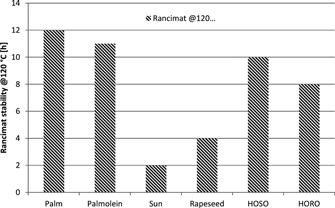
Rancimat stability at 120°C of different oils and fats. Fats with a higher level of saturated fatty acids (palm oil/palmolein) and with a high level of oleic acid (high oleic sunflower oil (HOSO)/high oleic rapeseed oil (HORO)) show significantly higher values then sunflower and rapeseed oil, which have high levels of fatty acids with two or more double bonds.

In frying applications, for example palm olein IV 64 or palm olein IV 56 can very often be replaced by liquid oils. Still, also here the choice of the right liquid oil is important. Most liquid oils have a significant lower level of saturated fatty acids and a higher level of polyunsaturated fatty acids than palm oil. This gives them a positive reputation in respect of health, but also on the other hand, a lower stability in terms of oxidative degradation. Linoleic acid is ca. 40 times more reactive then oleic acid and linolenic acid is ca. 2.4 times more reactive than linoleic acid 3. Therefore, oils with a high level of saturated and/or monounsaturated fatty acids have a higher stability against oxidation than oils with a high level of fatty acids with two or more double bonds. **Figure** 3 shows the rancimat stability index of various oils. Due to its high level of saturated and monounsaturated fatty acids, palm oil and its fractions have a relatively high rancimat stability index and are therefore ideal for frying applications, where the fat is typically used at high temperatures of circa 180°C, these high temperature conditions promote oxidation of frying fats, especially in less stable oils. Conventional rapeseed oil and sunflower oil have a high level of polyunsaturated fatty acids, and therefore oxidize relatively quickly in the frying process. From the oxidation, breakdown products arise (compounds like aldehydes), which can even be regarded as unhealthy 4. The use of liquid oils that contain a high level of oleic acid is more appropriate to replace palm oil in frying applications. High oleic sunflower and high oleic rapeseed oil would both be good solutions as they have high levels of monounsaturated fatty acids and a rancimat stability index only slightly lower than palm oil or palm olein, due to their elevated levels of oleic acid and reduced levels of both linoleic and linolenic acid.

## 3‐MCPD‐ and glycidolesters

3‐MCPD‐ and glycidolesters are contaminants that are formed during the refining process, especially the deodorization step. Various researchers demonstrated that in traditionally processed palm oil, the levels of both 3‐MCPD and glycidolesters are significantly higher than in many other edible oils/fats 2, 5. Nonetheless, 3‐MCPD‐ and glycidolester levels in palm oil can be partially mitigated by the application of newly developed process steps. Although it might be correct that with traditional refining procedures the levels of 3‐MCPD‐ and glycidolesters can be higher in palm oil, these contaminants are not confined only to palm oil; amounts of 3‐MCPD‐ and glycidolesters also occur in the refining process of almost all other oils and fats, albeit at generally lower levels.

## Conclusion

There are, in some countries, strong preferences towards palm‐oil‐free products. These are mostly related to sustainability (although the subject is being seriously tackled in the palm oil industry) and/or to health, i.e. levels of saturated fatty acids and of 3‐MCPD and glycidolesters. Both saturated fatty acids and 3‐MCPD and glycidolesters can be mitigated through both functional blends and improved processing techniques. As discussed above, it is possible to replace palm oil by liquid oils, blends with exotic fats, or blends with fully hydrogenated liquid oils. This will cause some technological challenges, however these are not insurmountable.

Still, the question remains whether the exclusion of palm oil is actually fully viable or a necessary step. Fats like coconut oil or cocoa butter, which have a perceived health advantage over palm oil, have a similar or even higher level of saturated fatty acids. Also, sustainable palm oil is already commercially available and efforts are undertaken to improve further palm oil sustainability. With improved refining techniques, it is possible to produce palm oil and palm oil fractions with lower levels of 3‐MCPD‐ and glycidolesters. Admittedly, sustainable palm oil refined in such a way that the levels of 3‐MCPD and glycidylesters are mitigated will incur additional costs, but non‐palm oil alternatives, especially if they are based on exotic fats, will have an even higher cost.

The technologies for the production of non‐palm alternatives for virtually all product types exist, but sustainable and 3‐MCPD‐ and glycidolester‐mitigated palm oils are also becoming more and more available. Therefore, customers have a choice of solutions to meet their individual requirements.
